# Kidney function and daily emtricitabine/tenofovir disoproxil fumarate pre-exposure prophylaxis against HIV: results from the real-life multicentric demonstrative project *PrEP Brazil*

**DOI:** 10.1186/s12981-022-00437-4

**Published:** 2022-02-24

**Authors:** Karla Cristina Silva Petruccelli, Djane Clarys Baía-da-Silva, Fernando Val, Monica Santos Valões, Nadia Cubas-Vega, Alexandre Vilhena Silva-Neto, Vanderson Sampaio, Aline Alencar, Roberto Pecoits-Filho, Rodrigo Carvalho Moreira, Sandra Wagner Cardoso, Ronaldo I. Moreira, Iuri Costa Leite, José Valdez Madruga, Esper G. Kallas, Paulo R. Alencastro, Brenda Hoagland, Beatriz Grinsztejn, Valdiléa Gonçalves Veloso Santos, Marcus Vinícius Guimarães Lacerda

**Affiliations:** 1 Universidade Federal do Amazonas, Manaus, Brazil; 2 Universidade do Estado do Amazonas, Manaus, Brazil; 3 Fundação de Medicina Tropical Dr Heitor Vieira Dourado, Av Pedro Teixeira, 25, Manaus, Amazonas 69040-000 Brazil; 4 Instituto Leônidas and Maria Deane, Fiocruz, Manaus, Brazil; 5 Fundação de Vigilância em Saúde do Amazonas, Manaus, Brazil; 6 Pontifícia Universidade Católica do Paraná, Curitiba, Brazil; 7 Instituto Nacional de Infectologia Evandro Chagas (INI), Fiocruz, Rio de Janeiro, Brazil; 8 Centro de Referência e Treinamento em DST/AIDS, São Paulo, Brazil; 9 Hospital das Clínicas, Faculdade de Medicina, Universidade de São Paulo, São Paulo, Brazil; 10 Hospital Sanatório Partenon, SES/RS, Porto Alegre, Brazil

**Keywords:** PrEP, Kidney function, HIV

## Abstract

**Background:**

Pre-Exposure Prophylaxis (PrEP) has demonstrated efficacy in the reduction of sexually transmitted HIV infections. The prolonged use of tenofovir disoproxil fumarate (TDF) and emtricitabine (FTC) co-formulation (TDF/FTC), however, may result in augmented risk of renal toxicity. We aimed to evaluate changes in the estimated Glomerular Filtration Rate (eGFR) in a real-world population setting of participants enrolled in *PrEP Brazil*, a 48-week prospective, open-label, demonstration study to assess the feasibility of daily oral TDF/FTC used by men who have sex with men and transgender women at high-risk of HIV infection, all over 18 years old.

**Methods:**

Kidney function was assessed by serial measurement of serum creatinine and eGFR with the Modification of Diet in Renal Disease Study (MDRD) formula on weeks 4, 12, 24, 36 and 48. Adherence to PrEP was assessed by dosing TDF concentration in dried blood spots at weeks 4 and 48, measured by liquid chromatography-mass spectrometry or mass spectrometry.

**Results:**

Of 392 participants completing the 48-week follow-up protocol with TDF blood detectable levels and eGFR measures, 43.1% were young adults, of Caucasian ethnic background (57.9%), with BMI below 30 kg/m^2^, without arterial hypertension. At screening, median eGFR was 93.0 mL/min/1.73 m^2^. At week 4 follow-up, 90 (23% of the study population) participants presented reductions in eGFR greater than 10 mL/min/1.73 m^2^ as compared to baseline eGFR, some as large as 59 mL/min/1.73 m^2^, but with no clinical outcomes (adverse events and renal adverse events) severe enough to demand TDF/FTC discontinuation. A negative relationship was observed between TDF blood levels and eGFR at weeks 4 (r = − 0.005; p < 0.01) and 48 (r = − 0.006; p < 0.01).

**Conclusions:**

These results suggest that the renal function profile in individuals on TDF/FTC may be assessed on week 4 and then only annually, allowing a more flexible medical follow-up in primary care centers.

**Supplementary Information:**

The online version contains supplementary material available at 10.1186/s12981-022-00437-4.

## Introduction

Although major advances have been made in HIV control worldwide, key populations remain at increased risk for HIV, demanding new prevention strategies [1]. The efficacy of oral pre-exposure prophylaxis (PrEP) with tenofovir (TDF)-based antiviral medication to prevent the acquisition of HIV by people living without HIV has been shown in randomized controlled trials across settings and populations [2]. Although TDF-based oral PrEP is safe and generally well-tolerated, some studies have found a statistically significant increase in risk of renal adverse events, while others have not [3]. In 2018, the *PrEP Brazil* study (ClinicalTrials.gov, number NCT01989611) showed that daily oral PrEP with TDF/FTC, delivered at no cost to men who have sex with men (MSM) and transgender women in three different Brazilian sites for 48 weeks, was feasible in real-world settings as part of the country’s public health system [4]. Overall, at 48 weeks, adherence to TDF/FTC was high, with 74% showing TDF levels expected to confer protection, with only two seroconversions in patients with undetectable levels of TDF in dried blood spot (DBS) [4].

TDF may induce mitochondrial damage in renal proximal tubular cells, leading to renal dysfunctions such as glucosuria, tubular proteinuria, and urinary electrolyte disturbances [5]. There may also be a reduction in the glomerular filtration rate (GFR) when measured by creatinine clearance, which can either stabilize after 4 weeks without further deterioration [6] or lead to progressive damage in kidney function [7]. The risk of kidney injury appears to be cumulative and linked to TDF exposure, making individuals in PrEP programs a population at potential risk, requiring routine monitoring of glomerular and tubular function [8, 9]. Most studies on renal safety in PrEP populations address GFR reductions reaching levels below the safety threshold of 60 mL/min/1.73 m^2^ [10]. There is little documentation on GFR trajectories that do not reach such low levels, even with losses at proportional rates as high as 90 mL/min/1.73 m^2^, and also few reports on eventual long-term chronic kidney disease (CKD) risk in such cases [3]. In this multicenter, open-label study we analyzed the renal safety of daily oral TDF/FTC used as PrEP among participants at high risk of HIV infection over a follow-up period of 48 weeks, describing the effects of the use of TDF/FTC on GFR to enable a less burdensome follow-up routine in a real-life setting.

## Methods

### Study design and participants

This was a retrospective analysis of data collected in the *PrEP Brazil* study aiming to characterize the dynamics of creatinine as a surrogate marker of kidney dysfunction and the estimated GFR (eGFR) among included participants. Details of the *PrEP Brazil* study protocol can be found elsewhere [11]. Briefly, it was a 48 week, open-label, demonstration study to assess PrEP delivery in real-world settings, originally designed to be held in five different Brazilian referral centers for HIV prevention and care in Rio de Janeiro (*Fundação Oswaldo Cruz*), São Paulo (*Universidade de São Paulo* and *Centro de Referência e Treinamento em IST e AIDS*), Manaus (*Fundação de Medicina Tropical Dr Heitor Vieira Dourado*), and Porto Alegre (*Hospital Partenon*). PrEP containing TDF/FTC was provided at no cost to MSM and transgender women who were not living with HIV, 18 years or older, with GFR ≥ 60 mL/min/1.73 m^2^ and reported one or more sexual risk criteria in the previous 12 months. eGFR was measured at weeks 4, 12, 24, 36 and 48. Adherence to PrEP was assessed by dosing TDF concentration in dried blood spots at weeks 4 and 48. Only patients in whom tests for GFR measurement were performed in each of the five visits of the 48 week follow-up period and who had a drug concentration greater than 0 at follow-up weeks 4 and 48 were included in the present analysis.

### Ethics

*PrEP Brazil* protocol was approved by the Brazilian National Ethics Review Board (CONEP) (CAAE 08405912.9.1001.5262), and by all the individual sites’ boards.

### Procedures

After enrollment participants were scheduled for follow-up visits at 4 weeks, 12 weeks, and every 12 weeks thereafter. Serum creatinine was measured locally at baseline and all weeks. Demographic data and sexual risk inclusion criteria were collected during pre-screening and screening (baseline) visits. Participants responded to computer-assisted self-interviews on registration and all study visits [11]. At every visit, HIV serological testing was performed according to the Brazilian Ministry of Health algorithm [12], as well as individual HIV RNA as per protocol. One positive test was followed by other different rapid tests in a sequential manner, and viral presence was confirmed by the HIV viral load quantification test [13].

DBS were collected for assessment of TDF and FTC levels from all participants at weeks 4 and 48 and were measured through liquid chromatography-mass spectrometry or mass spectrometry at the University of Colorado Antiviral Pharmacology Laboratory (Denver, CO, EUA) according to standard procedures [14]. Creatinine measurements were performed using the Jaffé method [15]. Modification of Diet in Renal Disease Study (MDRD) [16] was used to estimate GFR. CKD-Epi calculation was not part of the PrEP Brazil original protocol. Participants were also submitted to urinalysis with dipstick testing for the presence of protein. Baseline measurements occurred before PrEP start. Participants were stratified according to eGFR values into: (1) some abnormal kidney function (eGFR ≤ 90 mL/min/1.73 m^2^, group 1) and (2) normal function (eGFR > 90 mL/min/1.73 m^2^, group 2) based on thresholds defined by *Kidney Disease: Improving Global Outcomes* (KDIGO) for CKD [17]. Body Mass Index (BMI) and blood pressure were categorized according to the World Health Consultation on Obesity and to the 2017 American Heart Association Guidelines, respectively [18, 19].

### Statistical analysis

Fisher’s exact test or qui-squared test were used to compare distributions between groups, as appropriate. ANOVA and post hoc analysis by Bonferroni were used to compare filtration rates between weeks. Logistic regression was used to estimate odds ratios and respective confidence intervals to identify associations between renal function and clinical variables. Linear regression was performed for the continuous variables (eGFR, TDF concentration and eGFR variation). Statistical analysis was performed using Stata^®^ version 13 (Stata Corporation, College Station, TX, USA).

## Results

**Fig. 1:**
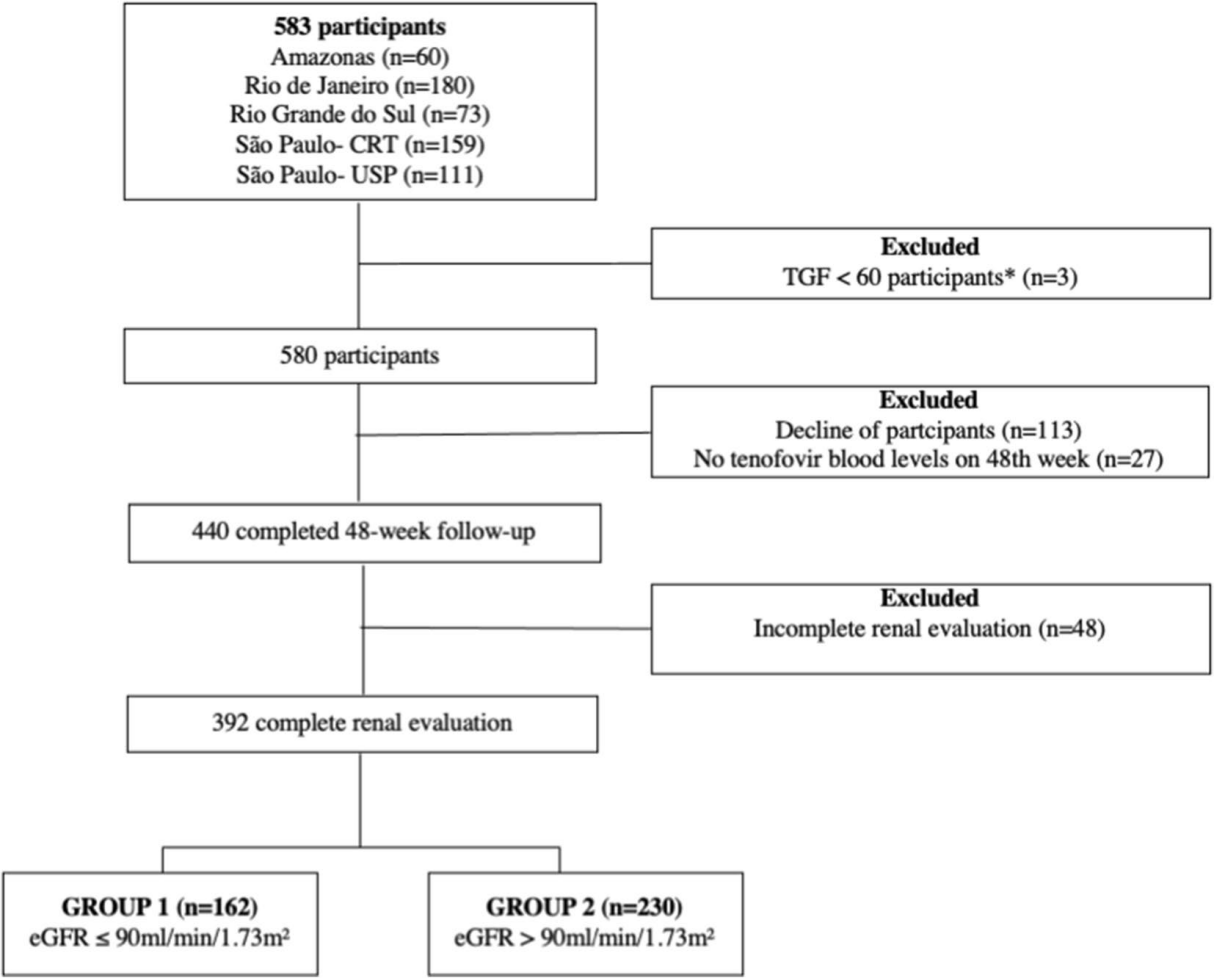
Study flowchart of *PrEP Brazil* study enrolled patients

Five hundred eighty-three participants were included in the *PrEP Brazil* study, of which 440 (75.9%) completed the week 48 follow-up visit, from June 4, 2014, to December 13, 2016. Out of these, 392 (67.6%) had all eGFR measurements and a drug concentration greater than 0 at follow-up weeks 4 and 48 (Fig. 1). Most participants (43.1%) were young adults, of Caucasian ethnic background (57.9%), with BMI below 30 kg/m^2^, without arterial hypertension history (Table 1), and with median eGFR at baseline of 94 mL/min/1.73 m^2^ (IQR = 82–107). Compared with participants with normal renal function, those with abnormal kidney function were older (11.7% vs 1.7%) without any other significant differences respective to clinical or laboratory measurements.

**Table 1:** Demographic and clinical characteristics of patients at inclusion visit

	eGFR ≤ 90* n = 162	eGFR > 90* n = 230	Total* n = 392	P
Age groups (in years)				
18–27	36 (22.2)	119 (51.7)	155 (39.5)	< 0.001
28–37	80 (49.4)	89 (38.7)	169 (43.1)	
38–47	26 (16.0)	18 (7.8)	44 (11.2)	
48–57	19 (11.7)	4 (1.7)	23 (5.9)	
> 57	1 (0.6)	0 (0.0)	1 (0.3)	
Ethnic background				
Caucasian	99 (61.1)	128 (55.7)	227 (57.9)	0.77
Afro-descendant	17 (10.5)	27 (11.7)	44 (11.2)	
Mixed race	45 (27.0)	72 (31.3)	117 (29.8)	
Indigenous	0 (0.0)	1 (0.4)	1 (0.3)	
Asian	1 (0.6%)	2 (0.9)	3 (0.9)	
BMI^1^ category (kg/m^2^)				
Underweight^a^	3 (1.9)	7 (3.0)	10 (2.6)	0.59
Normal^b^	69 (42.6)	117 (50.9)	186 (47.4)	
Overweight^c^	68 (42.0)	77 (33.5)	145 (37.0)	
Obesity class 1^d^	16 (9.9)	21 (9.1)	37 (9.4)	
Obesity class 2^e^	3 (1.9)	4 (1.7)	7 (1.8)	
Obesity class 3^f^	0 (0.0)	1 (0.4)	1 (0.3)	
Not measured	3 (1.8)	3 (1.3)	6 (1.5)	
Blood pressure (BP) category				
Normal^g^	101 (62.3)	131 (57)	232 (59.2)	0.64
High normal^h^	44 (27.2)	75 (32.6)	119 (30.4)	
Hypertension stage 1^i^	13 (8.0)	20 (8.7)	33 (8.4)	
Hypertension stage 2^j^	4 (2.5)	4 (1.7)	8 (2.0)	
Semi-quantitative proteinuria				
Absent	120 (74.1)	172 (74.8)	292 (74.5)	0.85
30–100 mg/dL	12 (7.4)	14 (5.7)	26 (6.4)	
100–300 mg/dL	2 (1.2)	4 (1.7)	6 (1.5)	
300–1000 mg/dL	1 (0.6)	1 (0.4)	2 (0.5)	
> 1000 mg/dL	0 (0.0)	1 (0.4)	1 (0.3)	
Not measured	27 (16.7)	39 (17.0)	66 (16.8)	

*Data are presented as number of patients (n) followed by percentage in parenthesis (%) ^1^BMI: body mass index; ^2^eGFR: estimated glomerular filtration rate; ^a^BMI < 18.5; ^b^BMI 18.5–24.9; ^c^BMI 25.0–29.9; ^d^BMI 30.0–34.9; ^e^BMI 35.0–39.9; ^f^BMI ≥ 40; ^g^BP < 130/85; ^h^BP 130–139/85–89; ^i^BP 140–159/90–99; ^j^BP ≥ 160/ ≥ 100

**Fig. 2:**
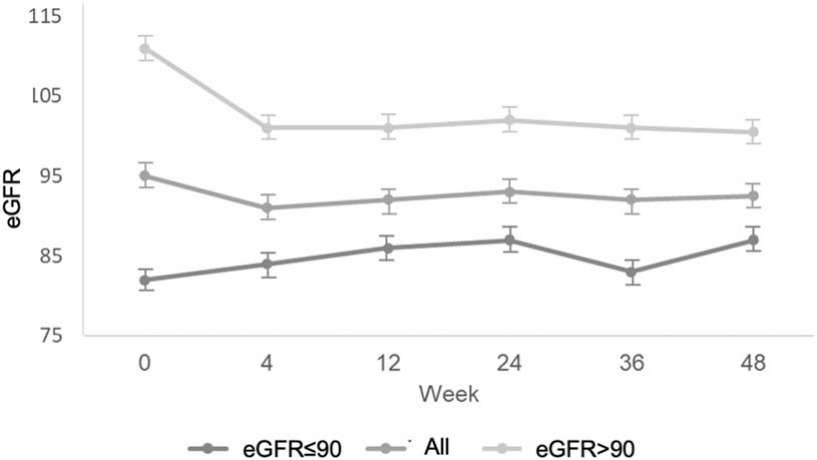
eGFR changes during the study period (48 weeks). Changes in eGFR over the follow-up period in relation to baseline in all analyzed patients are shown in the central line and in the subgroups of patients with eGFR ≤ 90 mL/min/1.73 m^2^ (n = 162) and eGFR > 90 mL/min/1.73 m^2^ (n = 230)

At week 4 follow-up visit, 90 (23.0%) participants experienced eGFR reductions greater than 10 mL/min/1.73 m^2^; 59 participants had eGFR reductions between 10 and 20 mL/min/1.73 m^2^, 19 showed reductions between 21 and 30 ml/min/1.73 m^2^ and 12 participants’ reductions were larger than 30 mL/min/1.73 m^2^. There was therefore a significant reduction in eGFR when compared to the baseline visit (eGFR reduction = − 3.46 ± 13.29; p < 0.001). Seven participants (1.8%) presented eGFR reductions higher than 30% from baseline. There was no statistical difference between the proportional variation of the eGFR observed at week 4 and subsequent visits. Participants with lower eGFR at baseline showed a significant increase, and those with normal eGFR at baseline a significant decrease overtime, as shown when comparing baseline and week 4 visit’s eGRF values (group 1, a gain of 2.13 units in eGFR, and group 2, loss of 7.34 units, p < 0.001) (Fig. 2). Both changes remained unaltered at subsequent visits (Additional file 1: Table S1). eGFR variation was adjusted for BMI, race, and proteinuria but there was no demonstrable association between these variables and the rate of eGFR variation (Additional file 1: Table S2).

Protocol violations were detected post-enrollment in three participants in whom PrEP was prescribed even with GFR < 60 mL/min/1.73 m^2^ (53, 54, and 57 mL/min/1.73 m^2^). Two of these participants adhered to PrEP and completed the entire follow-up. The participants’ kidney function remained stable throughout the entire follow-up, with improvement seen after week 4 on PrEP (77 and 72 mL/min/1.73 m^2^) and a slight decrease on week 48 (72 and 65 mL/min/1.73 m^2^ respectively). These individuals, however, were not included in the analyses.

**Fig. 3:**
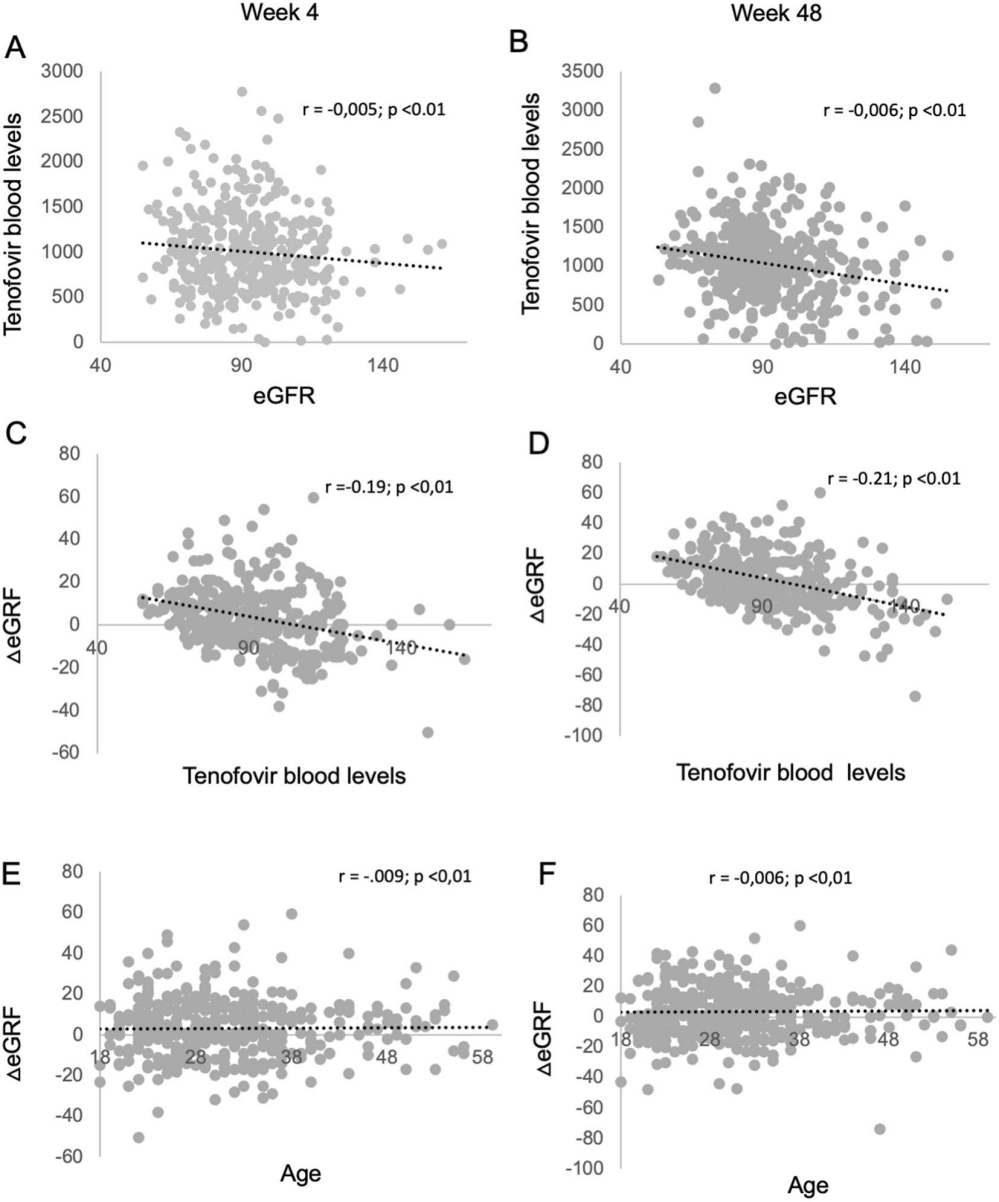
Tenofovir and eGFR correlations at week 4 and week 48. Correlation between tenofovir blood levels and eGFR are shown for week 4 (**A**) and week 48 (**B**); correlation between variation in eGFR and tenofovir blood levels are shown for week 4 (**C**) and week 48 (**D**); correlation between variation in eGFR and age are shown for week 4 (**E**) and week 48 (**F**)

A negative relationship was observed between TDF blood levels and eGFR at week 4 (r = − 0.005; p < 0.01) (Fig. 3A) and at week 48 (r = − 0.006; p < 0.01) (Fig. 3B). There was no relation between the proportion of eGFR loss and participants’ ethnic background (p = 0.291 at week 4; p = 0.527 at week 48), BMI (p = 0.311 at week 4; p = 0.729 at week 48), the prevalence of arterial hypertension (p = 0.162 at week 4; p = 0.585 at week 48), or incident proteinuria (p = 0.970 at week 4; p = 0.394 at week 48). There was an inverse relationship between the proportional reduction in eGFR and TDF blood levels at week 4 (r = − 0.19; p < 0.01) (Fig. 3C) and week 48 (r = − 0.21; p < 0.01) (Fig. 3D) and with age at week 4 (r = − 0.009; p < 0.01) (Fig. 3E) and week 48 (r = − 0.006; p < 0.01) (Fig. 3F). Clinical and laboratory data did not suggest Fanconi’s syndrome, and therefore no tubular proteins were analyzed. There were no clinical outcomes severe enough to demand TDF/FTC discontinuation.

## Discussion

Careful evaluation of the nephrotoxicity associated with the use of TDF/FTC in the context of PrEP for HIV used to be part of pharmacological surveillance strategies during the roll-out of this preventive intervention. In Brazil, PrEP became a public and free strategy of the combined HIV prevention, accessible for all, in December 2017. In this study, we assessed the association of PrEP with eGFR variation over 48 weeks using data from participants enrolled in the *PrEP Brazil* study. Grinsztejn et al. [4] showed that PrEP was feasible and safe, with very few adverse events sufficiently severe to lead to exclusion of participants due to a decline in renal function. Despite the lack of clinical manifestations, a significant and sustained variation in eGFR was recorded, similar to that reported in other studies regarding PrEP users [8, 20, 21]. In our study, eGFR reached its highest variation as early as week 4 of follow-up after the start of TDF/FTC-based PrEP, when compared to the baseline visit, and was maintained until the week 48 follow-up visit. This suggests a functional stabilization. The average loss of eGFR over the 48-week follow-up is similar to those found in the Intervention Préventive de l’Exposition aux Risques avec et pour les Gays (ANRS IPERGAY) [22] and Iniciativa Profilaxis Pre-Exposición Open Label Extension (iPrEx OLE) studies [21]. Moreover, epidemiological data suggest that significant renal impairment is uncommon in patients with long-term exposure to TDF/FTC [20].

Age was a risk factor for the pattern of kidney dysfunction in this group of volunteers. Older individuals had a higher frequency of baseline eGFR over 90 mL/min/1.73 m^2^ and had significantly greater eGFR losses after the intervention, in agreement with previous reports [8, 20, 21]. We did not observe an association between the degree of eGFR reduction and other traditional risk factors for renal dysfunction, such as arterial hypertension or obesity [22, 23]. In contrast to what has been observed in previous studies [22, 23], a higher frequency of eGFR reduction was observed amongst individuals who had higher baseline GFR values. The main CKD risk factors in the general population are diabetes, hypertension, older age, African-American origin, obesity, and prevalence of cardiovascular disease [24–26]. Many of these were either exclusion factors in the present study or were found not associated with a greater decrease of eGFR in the *PrEP Brazil* population.

The observed functional losses could be attributed to the use of the TDF/FTC-based combination for PrEP, however, slight reductions in GFR could also be interpreted as hemodynamic changes and not a reflection of toxicity. A reduction in GFR in the early period post drug initiation is seen in most kidney protective therapies, such as ACE, ARBs and SGLT2i [24, 26]. Mugwanya et al. [27] evaluated the safety of eGFR by monitoring groups of PrEP users semi-annually compared to the quarterly monitoring standard recommended by the WHO, and found no superiority of one routine over the other, adding that lower frequencies of control visits may provide gains in terms of adherence by users. The current results suggest that in low-risk groups, such as younger individuals and those with eGFR higher than 90 mL/min/1.73m^2^ without signs of proteinuria, broader monitoring intervals are possible without compromising safety, particularly in those patients with eGFR above 60 mL/min/1.73 m^2^ after 4 weeks of PrEP. One possibility is to apply the KDIGO CKD guidelines of follow-up [17] and monitor the kidney function in PrEP users annually. This reduction in the number of follow-up visits may improve adherence to PrEP protocols, and, in addition, allow better use of resources without impacting on standards of efficacy and safety, improving a particularly sensitive issue in low-income countries.

The present study had some limitations. The MDRD formula used for estimating GFR has a greater chance of bias when compared to the CKD-EPI formula, especially in groups whose GFR is greater than 60 mL/min/1.73 m^2^ [28]. The average exposure to TDF/FTC was 48 weeks, thus limiting our ability to predict the long-term effects on the kidneys, although the renal effects remained stable after the week 4 follow-up visit. Tenofovir interferes with tubular creatinine secretion, leading to a reduction of the accuracy of eGFR creatinine-based equations when compared to direct filtration by iothalamate clearance [29], making the use of formulas that add the dosage of cystatin C advisable [30]. Another limiting factor is the fact that the study is based on a healthy group of participants with no prevalent diseases potentially harmful to kidney function such as diabetes mellitus. Finally, there was no evaluation of tubular function, as described in previous studies [31], suggesting their evaluation in the same protocol to fully characterize renal dysfunction.

## Conclusions

In conclusion, even though here the use of the TDF/FTC combination caused a reduction in eGFR in healthy individuals, the use of PrEP was not a determinant of severe changes in renal function. Thus, in some subgroups of individuals, once the pattern of renal function behavior is established at the fourth week of PrEP, it may be monitored over a broader time interval visit schedule than the one currently recommended, demanding only yearly eGFR measurements instead of quarterly visits, without affecting protocol effectiveness or patient safety. This reduction in visit frequency because of the steady pattern of alteration in kidney function after week 4 may have a beneficial impact on public health management by significantly lowering health care costs and enhancing users’ adherence to PrEP oral regimens. Even with the emergence of other drug options such as tenofovir alfenamide, TDF/FTC may still remain a more feasible PrEP option, particularly in low-income countries. This schedule may also be applied to intermittent “On demand” PrEP programs, which have shown effectiveness in HIV protection with a reduced risk to kidney function [22], but still demand GFR regular monitoring.

## Supplementary Information

**Additional file 1: Table S1.** Glomerular filtration rate variation and week-by-week comparison by group. **Table S2.** Logistic regression to identify associations between eGRT variations and clinical variables.

## Data Availability

All data and materials are available.
