# Supplementary material for: Kidney function and daily emtricitabine/tenofovir disoproxil fumarate pre-exposure prophylaxis against HIV: results from the real-life multicentric demonstrative project PrEP Brazil
Source: AIDS Res Ther. 2022 Feb 24;19:12. doi: 10.1186/s12981-022-00437-4 (PMC8867642; doi:10.1186/s12981-022-00437-4)
Supplement: Supplementary file 1 — Additional file 1: Table S1. Glomerular filtration rate variation and week-by-week comparison by group. Table S2. Logistic regression to identify associations between eGRT variations and clinical variables. [file 12981_2022_437_MOESM1_ESM.docx]

**Additional Table S1:** Glomerular filtration rate variation and week-by-week comparison by group.

| Week | **All** | | | | | | | **Group 1** | | | | | | | | **Group 2** | | | | | | |
| --- | --- | --- | --- | --- | --- | --- | --- | --- | --- | --- | --- | --- | --- | --- | --- | --- | --- | --- | --- | --- | --- | --- |
|  | Median | Min | max | mean | sd | p | _95%_CI | Median | Min | max | mean | sd | p | _95%_CI | Median | | min | max | mean | sd | p | 95% CI |
| 4 vs baseline | -3 | -49 | 38 | -3.46 | 13.29 | **0.000** | -5.74 to -1.18 | 1 | -21 | 38 | 2.13 | 10.83 | **0.000** | -5.74 to -1.18 | -7 | | -49 | 23 | -7.34 | 13.48 | **0.000** | -10.39 to -4.30 |
| 12 vs baseline | -2 | -41 | 45 | -2.96 | 14.20 | **0.002** | -5.24 to -0.68 | 2 | -18 | 45 | 3.74 | 12.00 | **0.000** | -5.24 to -0.68 | -8,5 | | -41 | 43 | -7.63 | 13.75 | **0.000** | -10.67 to -4.59 |
| 24 vs baseline | -1 | -45 | 41 | -2.07 | 14.89 | **0.114** | -4.35 to 0.21 | 3 | -30 | 41 | 4.32 | 13.26 | 0.110 | -4.35 to 0.21 | -5 | | -45 | 35 | -6.52 | 14.37 | **0.000** | -9.57 to -3.48 |
| 36 vs baseline | -2 | -52 | 49 | -3.31 | 14.53 | **0.000** | -5.59 to -1.03 | 0 | -23 | 30 | 2.66 | 10.58 | **0.000** | -5.59 to -1.03 | -9,5 | | -52 | 49 | -7.46 | 15.46 | **0.000** | -10.50 to -4.42 |
| 48 vs baseline | -2 | -44 | 74 | -3.16 | 16.11 | **0.001** | -5.44 to -0.87 | 3 | -23 | 74 | 3.09 | 14.22 | **0.000** | -5.43 to -0.87 | -10 | | -10 | -44 | -7.50 | 15.95 | **0.000** | -10.54 to -4.46 |
| 12 vs 4 | 0 | -38 | 47 | 0.49 | 12.89 | 1.000 | -1.79 to 2.77 | 0 | -27 | 40 | 1.61 | 11.44 | 1.000 | -1.78 to 2.77 | 0 | | -38 | 47 | -0.29 | 13.80 | 1.000 | -3.33 to 2.76 |
| 24 vs 4 | 0 | -40 | 56 | 1.38 | 13.57 | 1.000 | -0.89 to 3.66 | 1 | -38 | 50 | 2.19 | 13.04 | 1.000 | -0.89 to 3.66 | 0 | | -40 | 56 | 0.82 | 13.92 | 1.000 | -2.22 to 3.86 |
| 36 vs 4 | 0 | -34 | 50 | 0.15 | 13.82 | 1.000 | -2.13 to 2.43 | 0 | -34 | 30 | 0.54 | 11.77 | 1.000 | -2.13 to 2.43 | -1 | | -34 | 50 | -0.12 | 15.11 | 1.000 | -3.15 to 2.92 |
| 48 vs 4 | 0 | -38 | 77 | 0.30 | 14.30 | 1.000 | -1.98 to 2.58 | 0 | -35 | 77 | 0.97 | 14.71 | 1.000 | -1.98 to 2.58 | -1 | | -38 | 39 | -0.16 | 14.03 | 1.000 | -3.20 to 2.88 |
| 24 vs 12 | 0 | -40 | 58 | 0.89 | 13.20 | 1.000 | -1.39 to 3.17 | 0 | -34 | 58 | 0.58 | 12.89 | 1.000 | -1.39 to 3.17 | 0 | | -40 | 57 | 1.10 | 13.44 | 1.000 | -1.94 to 4.15 |
| 36 vs 12 | 0 | -45 | 36 | -0.34 | 12.29 | 1.000 | -2.62 to 1.94 | 0 | -45 | 25 | -1.08 | 10.44 | 1.000 | -2.62 to 1.94 | 0 | | -37 | 36 | 0.17 | 13.43 | 1.000 | -2.87 3 to.21 |
| 48 vs 12 | 0 | -48 | 82 | -0.19 | 15.11 | 1.000 | -2.48 to 2.09 | 0 | -35 | 82 | -0.65 | 14.85 | 1.000 | -2.48 to 2.09 | -1 | | -48 | 42 | 0.13 | 15.32 | 1.000 | -2.92 to 3.17 |
| 36 vs 24 | 0 | -48 | 38 | -1.23 | 13.60 | 1.000 | -3.51 to 1.05 | 0 | -48 | 28 | -1.66 | 12,07 | 1.000 | -3.51 to 1.05 | 0 | | -34 | 38 | -0.94 | 14.59 | 1.000 | -3.98 to 2.11 |
| 48 vs 24 | -1 | -47 | 77 | -1.08 | 14.74 | 1.000 | -3.36 to 1.20 | -1 | -47 | 77 | -1.23 | 14,97 | 1.000 | -3.36 to 1.20 | -1 | | -46 | 43 | -0.98 | 14.62 | 1.000 | -4.02 to 2.06 |
| 48 vs 36 | 0 | -40 | 83 | 0.15 | 14.12 | 1.000 | -2.13 to 2.43 | 0 | -28 | 83 | 0.43 | 13.76 | 1.000 | -2.13 to 2.43 | 0 | | -40 | 34 | -0.04 | 14.39 | 1.000 | -3.08 to 2.99 |

**Additional Table S2 -** Logistic regression to identify associations between eGRT variations and clinical variables.

|  | Univariate | | | Multivariate | | | | Adjusted multivariate | | | |
| --- | --- | --- | --- | --- | --- | --- | --- | --- | --- | --- | --- |
| EGF variation | OR | p | IC | OR | P | IC | | OR | p | IC | |
| eGFR>90 mL/min/1.73m | -11,8197 | 0 | -14.95459 -8.684872 | -11,716 | 0 | -14,998 | -8,433 | -13,453 | 0 | -16,406 | -10,499 |
| Age | -0,012 | 0 | -.2183196 -.1787627 | -0,219 | 0,038 | -0,426 | -0,013 | -0,239 | 0,011 | -0,424 | -0,054 |
| BMI | 1,298923 | 0,164 | -.5310337 3.12888 | 0,83 | 0,331 | -0,845 | 2,504 | 0,877 | 0,267 | -0,673 | 2,427 |
| Proteinuria | -0,07377 | 0,968 | -3.712658 3.565118 | -0,484 | 0,764 | -3,654 | 2,686 | - | - | - | - |
| Race | 1,353535 | 0,127 | -.3862236 3.093293 | 1,324 | 0,103 | -0,267 | 2,916 | 1,218 | 1,218 | -0,226 | 2,662 |
| Systemic blood pressure | 0,273806 | 0,811 | -1.970398 2.518009 | 0,619 | 0,568 | -1,513 | 2,751 | - | - | - | - |
